# Advances in microbial metagenomics and artificial intelligence analysis in forensic identification

**DOI:** 10.3389/fmicb.2022.1046733

**Published:** 2022-11-15

**Authors:** Qing He, Xueli Niu, Rui-Qun Qi, Min Liu

**Affiliations:** ^1^Department of Dermatology, The First Hospital of China Medical University, Shenyang, China; ^2^Key Laboratory of Immunodermatology, Ministry of Education and NHC, National Joint Engineering Research Center for Theranostics of Immunological Skin Diseases, Shenyang, China; ^3^Institute of Respiratory Disease, China Medical University, Shenyang, China

**Keywords:** artificial intelligence, microbiome, machine learning, forensic microbiology, forensic science, microbial forensics

## Abstract

Microorganisms, which are widely distributed in nature and human body, show unique application value in forensic identification. Recent advances in high-throughput sequencing technology and significant reductions in analysis costs have markedly promoted the development of forensic microbiology and metagenomics. The rapid progression of artificial intelligence (AI) methods and computational approaches has shown their unique application value in forensics and their potential to address relevant forensic questions. Here, we summarize the current status of microbial metagenomics and AI analysis in forensic microbiology, including postmortem interval inference, individual identification, geolocation, and tissue/fluid identification.

## Introduction

“Microorganism” is a general term for tiny organisms that exist in nature, mainly including bacteria, viruses, and fungi, which are invisible to the naked eye or cannot be observed clearly. Microorganisms are small, simple in structure, and widely present in nature and the human body. The microbiome and metagenomics are rapidly emerging due to the progress of genome sequencing technology, improved microbial sampling methods and the rise of bioinformatics. In the era of big data, artificial intelligence and its related technologies continue to be developed and innovated, and corresponding results have been widely used in many disciplines, including forensics (Rahaman et al., 2020; Zhang et al., 2021a; Chen et al., 2022).

## Postmortem interval estimation

Inference of the time of death, or the postmortem interval (PMI), is an important task during forensic examination. Host- and environment-related microbial community succession during postmortem decay, which occurs in a regular, clock-like manner after human death, provides novel ideas for PMI inference (Metcalf et al., 2016). Johnson et al. (2016) sampled the skin microbiota in the nasal and ear canals of decomposing human cadavers to establish an algorithm for predicting the PMI, and thereby successfully demonstrated that the skin microbiota is a promising tool in forensic death investigations. The application of microbial community changes for PMI estimation has gradually become a topic of major interest in forensic research.

The oral cavity is one of the key research fields of human microbial communities, and its microbial community richness is one of the most abundant areas and the second largest human complex after gastrointestinal tract. Adserias-Garriga et al. (2017) monitored the oral microbiota of donated human bodies within 12 days after death. They found that Firmicutes and Actinobacteria are the predominant phyla in the fresh stage, Tenericutes is the predominant phyla in bloat stage, and Firmicutes is the predominant phyla in advanced decay. Dong et al. (2019) found that when the PMI was 0 h, the dominant phyla in the oral cavity of mice were Proteobacteria, Firmicutes, Actinobacteria, and Bacteroidetes. Within 240 h after the death of mice, the Proteobacteria and Firmicutes always occupied the dominant position. The oral microbiota changes in mice are different from those in human decaying bodies. By constructing linear regression models between relative abundance and postmortem intervals, Gamma-proteobacteria and Proteus species were the best candidates for use to infer the PMI, especially the late PMI. The *R*^2^ value of both constructed linear models was 0.99.

Microorganisms play a vital role in the decomposition process. However, relatively few studies are available on the postmortem migration behavior of microbial communities inside cadavers. Liu et al. (2020) assessed the microbial community structure in the brain, heart, and cecum of mice at 15 d postmortem and found that an artificial neural network (ANN) combined with the postmortem microbial dataset from the cecum was the optimal model; mean absolute error of 1.5 ± 0.8 h within 24-h decomposition and 14.5 ± 4.4 h within 15-day decomposition. This model is potential to serve as an advantageous technique in PMI inference, however, further verification is needed.

The above studies exposed cadavers to the air during decomposition. However, the microbial community in buried decomposing cadavers may be different from that in cadavers exposed to air due to different conditions such as oxygen content, humidity, light, and soil composition. Zhang et al. (2021b) analyzed postmortem microorganisms in the gravesoil, rectum, and skin of buried rats using the random forest algorithm to predict the PMI. The results showed that the predicted MAEs of the microorganisms in the rectum, cadaver skin, and gravesoil were 2.06, 2.13, and 1.82 days, respectively, within 60 days after death. This study developed the first model to predict the PMI based on microbial community succession and machine learning algorithms for buried bodies, which can provide information on the timing of buried body cases for forensic investigations.

Deel et al. (2021) placed six human donor subjects remains outdoors to decompose on the soil surface, with three samples each placed in spring and summer. Microorganisms in the skin and soil can naturally decompose the corpse to expose the ribs. The investigators developed a PMI prediction model using colonies on ribs in combination with the random forest algorithm. The accuracy of PMI prediction within 9 months was approximately (±34) days. This study represents a preliminary attempt to study the continuity of microbial communities in postmortem corpse remains, which may provide a tool for forensic investigators to estimate the time since death of skeletal remains. However, limitations remain, such as small sample sizes, differences between seasons, including differences in soil moisture, inorganic salts, and microbial contents, and variations in the organic composition of bones and other skeletal degradation indicators.

## Individual identification

Individual identification is one of the most important tasks in forensic science. A number of studies have shown that microecosystems such as the skin, oral cavity, and intestine have obvious polymorphisms and individual differences. The differences in microbial community composition and abundance in human microecosystems constitute the basis of microbial use for individual identification. In theory, each individual carries a unique set of microorganisms that differs from those of other individuals, which can be identified through microbiome analysis, and this particular microbial community can persist over long periods. Therefore, microbiome characterization is potentially applicable to forensic human identification.

Franzosa et al. found that the microbiome of an individual can specifically identify its source host in a population of more than 100 people, the performance of the gut microbiome is very stable, and more than 80% of individuals can still be accurately located after 1 year (Franzosa et al., 2015). Another study found that the genotypic composition of the 16S rRNA of Cutibacterium acnes is individual-specific. The random forest machine learning method was used to combine the 16S rRNA genotype of C. acnes with the skin microbiome profile data, and the accuracy of individual identification was ~90% (Yang et al., 2019). Over time, the 16S rRNA genotype of C. acnes was more stable than that of the skin microbiome profile.

The Budowle team conducted a series of studies on the application of forensic individual identification using skin microorganisms (Schmedes et al., 2017, 2018). The core microbiota of the skin was determined, and clade-specific markers were identified. A novel targeted sequencing panel, the hidSkinPlex, was developed, which contains 286 markers covering a range of taxonomies of specific microorganisms that are in high abundance on the human skin. Schmedes et al., (2018) achieved accuracy rates between 54.20% and 100.00% when classifying eight individuals with samples from three body sites (i.e., foot, hand and manubrium) by using regularized multinomial logistic regression and 1-nearest-neighbor classification. Woerner et al. (2019) used the same panel to classify 51 individuals across three body sites with nearest neighbor machine learning approaches. The accuracy rates of using phylogenetic distance or nucleotide diversity were 78.00% and 83.70%, respectively. As the number of individuals increased, the classification accuracy decreased.

Sherier et al. (2021, 2022) proposed that single nucleotide polymorphism genetic markers are more individualized than taxonomic markers. They designed an improved “hidSkinPlex+” system, which comprises 365 SNPs residing in 135 markers, fewer markers than the original hidSkinPlex. Eliminating the markers that do not contribute to classification accuracy can improve the enrichment process and increase the efficiency of machine learning. They reanalyzed the same sequencing data as those in Woerner et al. (2019), and found that the highest Wright’s fixation index (F_ST_) combined with support vector machine (SVM) could achieve higher accuracy in individual identification (*p* = 0.03, chi-squared test).

## Tissue/fluid identification

During forensic reconstruction of crime scene activities, identification of biological traces and their bodily origin provides valuable evidence that can be presented in court. However, traces and stains at the crime scene are often exposed to the environment outside the human body for a period before being processed in the laboratory. Dobay et al. (2019) detected some characteristic microorganisms with high abundance in semen, saliva, vaginal secretions, menstrual blood, peripheral blood, and skin. The study found that samples with 30 days of indoor exposure still harbor a microbial signature that can be used to identify bodily origins. The dominant microbial signature in skin, saliva, semen are Propionibacterium, Prevotella, and Bacteroides, respectively. Vaginal fluid and menstrual blood share their microbial signatures, as Lactobacillus makes up on average 75% and 86% of the bacterial reads. Hanssen et al. (2017) used standard pattern recognition based on principal component analysis in combination with linear discriminant analysis and found that the microbial community was well differentiated between saliva and skin, and the saliva microorganisms of different individuals have specificity. The accuracy of cross-validation was 94%. Based on massively parallel sequencing of the microbiome, Díez López et al. achieved accurate tissue-type classification of skin, saliva, and vaginal secretions by using taxonomy-independent deep learning networks (Díez López et al., 2019). Body-site classification accuracy of these test samples was very high as indicated by AUC values of 0.99 for skin, 0.99 for oral, and 1 for vaginal secretion. It can also provide forensically relevant blood samples (e.g., menstrual blood, nasal blood, fingertip blood, and venous blood) with accurate information about the source of blood in the body (Díez López et al., 2020). By analyzing the sequencing data of different body parts and soil mixture samples, Tackmann et al. (2018) identified a core set of ecologically informed microbial biomarkers for human body sites. Using Generalized Local Learning, 635 operational taxonomic units (OTUs) were reported as biomarkers, between 92 (nostril) and 326 (skin). Bacteroidetes, Firmicutes, Proteobacteria, and Actinobacteria were dominant in all investigated body sites. They found high fractions of positive Firmicutes and Bacteroidetes biomarkers in feces and Proteobacteria biomarkers in skin.

## Geolocation

The International Metagenomics and Metadesign of Subways and Urban Biomes (MetaSUB), which was launched in 2015, is a global network of scientists and clinicians developing knowledge of urban microbiomes by studying mass transit systems, the built environment, and hospitals. In forensic casework, a link is evident between a crime scene investigation, the suspect, and an object, location, or victim. The study of environmental metagenomics also introduces potential for new forensic applications such as geographical identification.

Researchers and volunteers of MetaSUB Consortium collected ~5,000 samples from the mass transit systems of 60 cities around the world. Analysis was performed using next-generation sequencing and genome sequencing technology, and the largest set of global urban microbial metagenomics research results to date was reported (Danko et al., 2021). Public data from MetaSUB Consortium were used by multiple research teams to perform geographic origin inference by using various bioinformatics and artificial intelligence algorithms. Huang et al. (2020) extracted features from metagenomic abundance profiles. By using logistic regression with L2 normalization, the prediction accuracy of the model reached 86% to infer city affiliation. Walker and Datta (2019) analyzed whole-genome sequenced microbiota sampled from 12 cities in seven different countries. The authors applied machine learning techniques to identify the geographical provenance of the microbiome samples. Up to 90% of the samples were correctly classified, demonstrating the potential of machine learning applications in biogeography, although further evidence is necessary to extend these applications to an evidentiary context. Ryan (2019) constructed a random forest classifier based on a dataset of 311 urban microbiome samples and correctly classified 83.3% of the samples.

## Conclusion

Microorganisms are widely distributed in nature and the human body. Microbial traces from the human body or crime scenes can be effectively used in forensic medicine to solve crime problems, showing huge potential and unique application value in forensic medicine. The rapid progress of artificial intelligence and its related technologies has markedly promoted the development of forensic microbiology, which has introduced novel ideas and tools for solving the problems in forensic practice. Research is still at the preliminary stage, and many challenges need further addressed, for example, limited sample sizes, model accuracies, unrealistic environmental settings, etc. As artificial intelligence analysis in forensic identification is novel innovation, there are only limited relevant research reports. Many researchers conducted a single study from a single perspective, and there was insufficient data to cross-verify the accuracy of these results. Although we have summarized these reports, it is not known how accurate the studies are. Nonetheless, it is foreseeable that microbiome-based evidence could contribute to forensic investigations in the future.

## Author contributions

QH: searched and analyzed the published literature, drafted the manuscript. XN: searched and analyzed the published literature. R-QQ and ML: reviewed and edited the manuscript. All authors contributed to the article and approved the submitted version.

## Funding

This study was funded and supported by National Natural Science Foundation of China (82173401 and U1908206).

## Conflict of interest

The authors declare that the research was conducted in the absence of any commercial or financial relationships that could be construed as a potential conflict of interest.

## Publisher’s note

All claims expressed in this article are solely those of the authors and do not necessarily represent those of their affiliated organizations, or those of the publisher, the editors and the reviewers. Any product that may be evaluated in this article, or claim that may be made by its manufacturer, is not guaranteed or endorsed by the publisher.
